# Pharmacological Significance and Phytochemical Diversity of the Genus *Lilium*: A Special Emphasis on the Himalayan *Lilium polyphyllum*

**DOI:** 10.3390/plants15142214

**Published:** 2026-07-20

**Authors:** Shafiq Ur Rahman, Haider Ali, Umair Sayad, Abid Ullah, Jafar Saifullah, Sultan Mehtap Büyüker, Atif Ali Khan Khalil

**Affiliations:** 1 Department of Pharmacy, Shaheed Benazir Bhutto University, Sheringal, Dir 18050, Khyber Pakhtunkhwa, Pakistan; shafiq@sbbu.edu.pk (S.U.R.); haiderpharmacist@gmail.com (H.A.); umairsayad91@gmail.com (U.S.); abid@sbbu.edu.pk (A.U.); 2 H.E.J Research Institute of Chemistry, International Center for Chemical and Biological Sciences, University of Karachi, Karachi 75270, Sindh, Pakistan; jafarchemi@gmail.com; 3 Department of Pharmaceutical Toxicology, School of Pharmacy, Medipol University, Istanbul 34810, Turkey; sultan.buyuker@medipol.edu.tr; 4 Department of Biotechnology, Yeungnam University, Gyeongsan 38541, Republic of Korea

**Keywords:** phytochemicals, ethnopharmacology, pharmacological potential, *L. polyphyllum*, conservation of endemic plants

## Abstract

The genus *Lilium* (Family Liliaceae), comprising economically important ornamental and medicinal perennial herbaceous plants, has gained increasing scientific interest due to its rich phytochemical diversity and broad spectrum of pharmacological activities. Several *Lilium* species have been traditionally utilized in various medicinal systems for the treatment of respiratory, inflammatory, gastrointestinal and neurological disorders. Among them, *Lilium polyphyllum* D. Don ex Royle is recognized as one of the beneficial medicinal species because of its ethnopharmacological importance and diverse bioactive constituents. Phytochemical investigations of *Lilium* species have identified numerous secondary metabolites, including flavonoids, phenolic compounds, steroidal saponins, polysaccharides, alkaloids and terpenoids, which contribute to their pharmacological potential. Recent pharmacological developments in the genus *Lilium* highlight the therapeutic potential of its steroidal saponins, alkaloids and polysaccharides, particularly regarding their action as immunomodulatory, anti-inflammatory and anti-tumor. While the isolated phytochemicals have not yet transitioned to markets, *Lilium* extracts are actively commercialized globally within regulated traditional medicine formulations. This review provides a comprehensive and updated overview of the botanical characteristics, phytochemical composition, pharmacological activities and therapeutic potential of the genus *Lilium*, with particular emphasis on *L. polyphyllum*. Furthermore, current knowledge gaps, including limited mechanistic studies, pharmacokinetic evaluations and clinical investigations, are discussed. The review also highlights the urgent need for conservation strategies, sustainable cultivation practices and biotechnological approaches such as plant tissue culture to protect natural populations of *L. polyphyllum* while ensuring a reliable supply of plant material for future pharmaceutical and nutraceutical development.

## 1. Introduction

The genus *Lilium* (Family Liliaceae) comprises 100–115 species and is a perennial, herbaceous, bulbous plant, widely recognized for its profound economic, ornamental, and ethno-medicinal value. The species are largely distributed across the temperate regions of the Northern Hemisphere. The diversity of these species is predominantly high in East Asia, with 55 species reported in China, followed by Japan (15 species), Korea (11 species) and 22 in North America, while the remaining are distributed in other areas [1,2,3]. Historical reports indicate that the cultivation of lilies in China dates back more than two millennia; however, systematic breeding programs were initiated in Japan and the US in the early 20th century, where they are cultivated for ethno-medicinal, ornamental, and cooking purposes. These breeding works focused on interspecific hybridization within the Sinomartagon section (*Lilium davidii*, *Lilium dauricum*, *Lilium lancifolium*, and *Lilium bulbiferum*) to develop commercially significant Asiatic and oriental hybrid groups [4]. While commercial cultivation of lilies has traditionally prioritized floral aesthetics [5,6], recent research has shifted considerably toward elucidating the phytochemistry and pharmacological potential of *Lilium* species [7,8,9].

**Figure 1:**
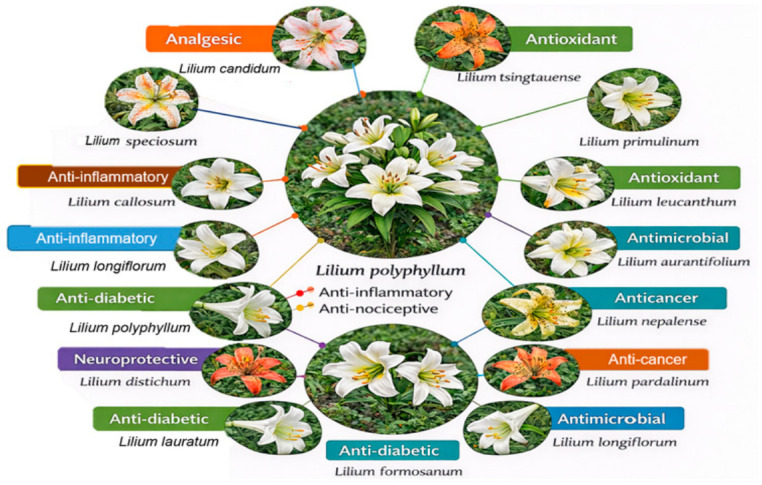
*Lilium* species recognized for their diverse pharmacological applications (compiled from the reported literature).

Species of the genus *Lilium* are morphologically distinguished by the presence of underground scaly bulbs. Phytochemical analysis has shown a diverse array of phytoconstituents within the genus, such as steroidal saponins, alkaloids, glycosides and polysaccharides, which contribute to its pharmacological potential in both traditional and modern medicine [9]. The diverse medicinal advantages of *Lilium* plants can be explained by the distinct and potent biological activities (Figure 1). The bulbs of *Lilium* species like *L. brownii* and *L. lancifolium* are widely utilized in traditional Chinese medicine (TCM) for the treatment of respiratory diseases and tumors and the management of insomnia and depression [10,11]. Current pharmacological investigations have provided scientific evidence of the traditional uses of some of the *Lilium* species, demonstrating the potent anti-inflammatory, immunomodulatory, anti-tumor, and cytoprotective properties across various in vitro and in vivo experimental models [9,12,13].

While many East Asian and European species of *Lilium* are significantly cultivated and chemically characterized, still, limited literature is available on *Lilium polyphyllum* D. Don (commonly known as white lily or kakoli, ksheerkakoli). Therefore, it represents a distinct phytochemical and conservation priority that requires a targeted scientific review. *L. polyphyllum* is endemic to the fragile alpine and sub-alpine habitats of the Indian Himalayan Region (IHR) at elevations between 1800 and 3700 m and is currently documented as a critically endangered species [14]. Unlike most of its abundant counterparts, the natural populations of this species have experienced marked declines due to a combination of strict ecological constraints, poor natural seed germination rates, and intense anthropogenic exploitation driven by the destructive harvesting of its underground bulbs [14,15,16].

Therapeutically, *L. polyphyllum* has a unique status and its bulbs are a core ingredient in over 30 Ayurvedic formulations, including Ashtavarga (a group of eight plants) valued for their anti-aging and vitality-enhancing properties [17,18,19]. Traditionally, this species is valued for treating intermittent fevers and sexual dysfunction. It also acts as an aphrodisiac, offers warmth in cold environments, and has anti-inflammatory, astringent, expectorant, and diuretic properties [20]. However, extensive application, increasing demand, and the limited availability of plant material have placed *L. polyphyllum* in an extremely vulnerable position.

To preserve this endangered genetic resource and meet commercial requirements sustainably, recent years have driven significant advancements in biotechnological interventions for *L. polyphyllum*. Optimized in vitro propagation approaches, such as somatic embryogenesis and bulb scale propagation, have been developed to bypass natural reproductive constraints and establish high-throughput conservation plans. Similarly, modern research has focused on optimizing the agronomic parameters necessary for the ex situ cultivation and domestication of this species outside its native Himalayan environments [19,21,22,23,24,25].

Although significant progress has been made in recent years, an integrated, comprehensive, updated evaluation that links the gap between the traditional importance of *L. polyphyllum* and modern scientific validation is still not available. This review provides a timely, systematic assessment of recent advances in cultivation, biotechnological micropropagation, phytochemistry, and preclinical evaluation of *L. polyphyllum* along with its generic species. Furthermore, this review aims to highlight key challenges associated with conservation and sustainable utilization, identifying future pharmacokinetic and clinical trials, and providing a framework for the biotechnology-driven conservation of this endangered Himalayan species. To ensure a balanced framework for this review, a structured literature search was conducted using specific bibliometric parameters as described in the following section.

## 2. Literature Search Methods

A comprehensive literature search was conducted using major academic databases and indexing platforms, including Scopus, PubMed, Web of Science, ScienceDirect, Wiley Online Library, and the academic search engine Google Scholar. The search strategy used combinations of keywords such as genus *Lilium*, *Lilium polyphyllum*, phytochemistry, secondary metabolites, pharmacological activities, antioxidants, antidiabetic, anti-inflammatory, anticancer, antibacterial, antifungal and cosmeceuticals, among others. The data collection/publications were not bound by a specific year range, focusing on articles published that satisfy the aim of the present review.

This study included and analyzed relevant peer-reviewed original research papers and systematic reviews focusing on the phytochemical characterization of phytoconstituents, preclinical in silico, in vitro, or in vivo experiments evaluating the efficacy, predicting molecular targets, safety, toxicities, clinical profile, along with traditional ethnomedicinal reports providing baseline medical records or formulation metrics, including Ayurvedic or traditional Chinese medicine. We excluded conference abstracts, patents, and brief perspectives without original or synthesized data. Data collected through this strategy forms the basis of the evaluation. A total of 163 occurrence records of *L. polyphyllum* were retrieved from GBIF. Records lacking geographic coordinates were excluded, leaving 36 georeferenced records. The coordinates were checked for validity and consistency with the reported countries, and duplicate coordinate pairs were removed. After quality control, 31 unique occurrence localities were retained for preparation of the distribution map. To better understand the implications of data, we first examine the broader taxonomic and botanical landscape of the genus *Lilium*, followed by a discussion of the isolated species profiles.

## 3. Representative Species of Genus *Lilium*

The *Lilium* species shows high diversity in its geographical distribution and morphological characteristics. As given in Table 1, the medicinally important species are present across diverse worldwide habitats, including temperate and alpine regions of Asia, the Middle East, Europe and North America. Due to the large geographical range, these species have gained different, distinct characteristics, particularly in their bulb appearance, stem behavior and floral morphology.

Some species, like *L. nepalense*, possess stoloniferous bulbs in comparison to others, which exhibit specialized rooting behaviors. Similarly, plants like *L. concolor* and *L. canadense* are characterized by adventitious roots arising above the bulbs [6]. In addition, aerial propagation mechanisms also differ; for instance, *L. lancifolium* and *L. bulbiferum* produce bulbils within their leaf axils [26]. Likewise, *L. candidum* produces a basal rosette of leaves during the winter months that senesces by the following summer, thus placing it in a separate growth cycle from other typical seasonal growth patterns as seen in other lilies like *L. longiflorum* [27]. Morphologies range from large fragrant, trumpet-shaped flowers of *L. regale*, *L. longiflorum*, and *L. auratum* to the highly reflexed, nodding petals of *L. henryi* and *L. pumilum*. Among the *Lilium* species, *L. polyphyllum* is distinguished by elongated, narrow white bulbs and white-to-light pink petals.

**Table 1:** A summary of high-value representative medicinal species within the genus *Lilium*, highlighting their common names, primary geographic distribution and specific morphological characteristics [28,29,30,31,32].

No	Species	Common Name	Geographical Origin	Specific Botanical Characteristics
1	*Lilium formosanum* A. Wallace	Formosan lily, Taiwan lily, Tiger lily	Taiwan	Bulb is small, rounded and reaches a diameter of around 2 to 4 cm. Stem is smooth to papillose with linear foliage.
2	*Lilium duchartrei* Franch	*Lilium* farreri	China, Arunachal Pradesh, Myanmar	Bulb is medium-sized, ovoid, whitish, with fleshy, overlap scales and the flowers are white with maroon spots.
3	*Lilium callosum* Siebold & Zuccarini		Southeast Asia	Bulb is flattened-globose around 15–30 mm in diameter and is made up of many imbricate, fleshy scales, without a tunic. Flowers are orange–red, narrow-petaled thrive in dry and sandy soils.
4	*Lilium nepalense* D. Don	Nepalese lily, Khiraule	Native to the Himalayas	Bulb is stoloniferous, while flowers are few, often solitary, pendant, and pale green.
5	*Lilium speciosum var. gloriosoides* Baker	*Japanese lily*, *Rubrum Lily*, *Spotted Lily*	Native to southern Japan and southern China, Korea	Bulb is medium to large with pale, loosely overlapping fleshy scales, while flowers are strongly scented.
6	*Lilium concolor* Salisbury	Morning star lily	China, Japan, Korea and Russia	Produces adventitious roots above the bulbs. Stems are cylindrical (terete), closely glabrous and smooth. Flowers are star-shaped with an orange–yellow to reddish color and are attractive.
7	*Lilium philadelphicum* Linnaeus var. *andinum*	Wood lily, Flame lily, Western red lily	Native to the East Coast to the Midwest of North America	Orange–red flowers, prefers dry and open habitats. Funnel-shaped, red–orange petals with brown spots near the base.
8	*Lilium leichtlinii* *subsp. maximowiczii* (Maxim.) J. Compton	Lily of the sun	Native to Japan	Yellow–orange flowers with black spots, graceful and heat-tolerant.
9	*Lilium regale* E. H. Wilson	Royal lily, King’s lily, Christmas lily, Trumpet lily	Native to the western Sichuan Province in southwestern China	Stems are rigid and leafy. Large, white, trumpet-shaped flowers.
10	*Lilium davidii* Duchartre ex Elwes	-	Indigenous to mountainous areas of Bhutan, Tibet, Arunachal Pradesh	Stem rooting lily (adventitious roots developing above the bulbs) also forms bulbils. Carries up to nearly 20 unscented flowers with recurved tepals.
11	*Lilium auratum* Lindl	Golden-rayed lily, Goldband lily	Japan	A dormant bulb that can be planted in spring or fall. White flowers with golden streaks and maroon spots, intensely fragrant.
12	*Lilium longiflorum* var. *scabrum* Masamune	Easter lily, White trumpet lily	Taiwan, Ryukyu Islands	Long white trumpet-shaped flowers, popular in floriculture. Long oval leaves and veins enter the horizontal direction.
13	*Lilium brownii* F. E. Brown ex Miellez	Hong Kong lily, Brown’s lily	China, Taiwan	Hermaphrodite has globose bulbs. Fragrant trumpet-shaped flowers with purple stripes.
14	*Lilium henryi* Baker	Henry’s lily, Gold Henryi	Central China	Leaves are lance-shaped and deep green, while flowers are orange with reflexed petals, tolerate heat and humidity.
15	*Lilium lancifolium* Thunb	-	China, Korea, Japan	Orange flowers with black spots, hardy and drought-tolerant.
16	*Lilium pumilum* Delile	Coral lily	Siberia, Mongolia, Korea	Red–orange nodding flowers with a compact growth habit.
17	*Lilium candidum* Linnaeus	Madonna lily	Native to the Middle East and South-Eastern Europe (Balkans)	Produces bulbs at ground level and, contrary to other lilies, grows a basal rosette of leaves during winter, which die the following summer. White trumpet-shaped flowers, known as the Madonna lily.
18	*Lilium polyphyllum* D. Don ex Royle	Himalayan lily, White lily	Himalayas (Pakistan, India, Nepal, Afghanistan)	Bulb is typically white, long and narrow and forms roots. Stems are a little stiff, while petals are a white or light pink color.

## 4. Traditional Uses of *Lilium* Species

**Figure 2:**
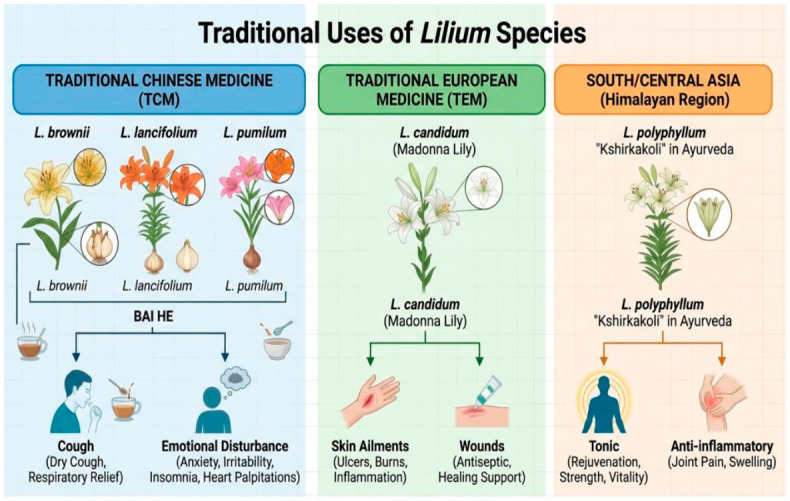
Traditional uses of *Lilium* species (compiled from the reported literature cited in Section 4).

For hundreds of years, the genus *Lilium* has been largely used in medicine, culture, and nutrition across Asia, Europe, and the Himalayas. Several species of *Lilium* have been incorporated into both local folk practices and formal conventional systems. Some of its important traditional uses are given in Figure 2. The bulbs of *L. brownii*, *L. lancifolium* and *L. pumilum* are referred to as “Bai He” in traditional Chinese medicine (TCM), which literally means “hundred unions” and represents healing and harmony. Classical TCM formulations such as “Bai He Gu Jin Tang” and “Bai He Zhi Mu Tang” include these species as key ingredients for treating chronic cough, palpitations, sleeplessness and emotional disturbance [10].

Traditional European Medicine (TEM) also capitalized on the *Lilium* species due to its considerable therapeutic potential, especially *L. candidum*, which has been used from ancient times for curing wounds and treatment of external burns, inflammatory skin ailments and ulcers. Extracts obtained from bulbs, roots, petals or leaves were frequently applied as ointments, infused oils or poultices to manage pain, tissue repair and reduce swelling [33].

One of the most valued medicinal bulbs in South and Central Asia, especially in the Himalayan region, is *Lilium polyphyllum* D. Don (*L. polyphyllum*), also known as “Kshirkakoli” in India. According to ethnobotanical data, it is used as a tonic, aphrodisiac, antipyretic, expectorant and anti-inflammatory [34]. Similarly, in East Asia, *L. lancifolium* bulbs and roots are used to treat respiratory disorders [1] while *L. pumilum* and *L. brownii* bulbs serve as food–medicine hybrids, which are boiled, sweetened or prepared into soups, especially for people who are ill or experiencing chronic fatigue [34].

## 5. Bioactivities of *Lilium* Species

**Figure 3:**
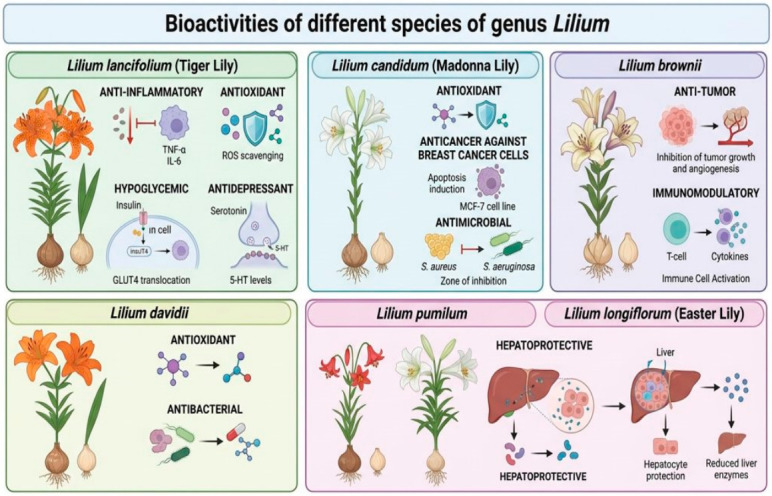
Bioactivities of *Lilium* species (compiled from the reported literature cited in Section 5 and Table 2).

There are numerous and diverse reported pharmacological activities of the genus *Lilium*, which are often species-specific. Some of the reported pharmacological activities along with species name, plant part and extract/compounds are given in Figure 3 and Table 2. *L. lancifolium* bulbs and root extracts have been noted for anti-inflammatory, antioxidant, hypoglycemic and antidepressant-like activities in different mouse models.

The bulbs of *L. candidum* possess antioxidant properties and have been noted for anticancer activity against human breast cancer cells (MCF-7). Similarly, the bulbs of *L. brownii* var. *viridulum* have anti-tumor and immunomodulatory potential due to the presence of steroidal saponins and polysaccharides.

In addition, polysaccharides from *L. davidii* var. *unicolor* roots and bulbs possess marked antioxidant and antibacterial effects. Hepatoprotective activity has also been noted in the bulbs of *L. pumilum* and *L. longiflorum* in animal models. The antimicrobial and antioxidant properties of *L. candidum* against *S. aureus* and *P. aeruginosa* have been confirmed by laboratory techniques, which confirmed their long-standing use as topical medications.

## 6. Current Progress, Translational Deficiencies, Marketed Formulations, Toxicity and Clinical Development

Despite significant development in the phytochemical characterization of the genus *Lilium*, its translation from its chemical diversity into pharmacological and industrial uses is a major challenge. The current literature has identified more than 100 distinct chemical metabolites; still, their pharmacological investigation and translation remain bottlenecked as the literature relies mainly on raw, unstandardized ethanol or aqueous extracts rather than purified, single-plant constituent monotherapies, resulting in significant analytical ambiguity. For instance, although individual metabolites such as regaloside A have revealed promising neuroprotective potential via the modulation of the BDNF/TrkB and PI3K/Akt/mTOR signaling cascades [58], the precise structure–activity relationships (SAR) analysis for the diverse array of *Lilium*-derived metabolites, including steroidal saponins, phenolics and phenylpropanoids identified and isolated from various species, has yet to be systematically established.

Furthermore, pharmacological investigations have evolved from descriptive phenotypic bioassays toward mechanism-based studies that elucidate the target-specific molecular basis of its bioactivity. Preclinical in vitro and in vivo models have focused on identifying specific molecular pathways, showing that the pharmacological effects of *Lilium*-derived fractions and highly branched functional polysaccharides can show significant anti-inflammatory activity via suppressing the production of pro-inflammatory cytokines, notably interleukin-6 (IL-6) and tumor necrosis factor-alpha (TNF-α), through the modulation of NF-κB and mitogen-activated protein kinase (MAPK) signaling [59]. In cancer, *Lilium*-derived constituents induce selective cytotoxic effects through the mitochondrial Bcl-2/Bax signaling pathway [60]. Additionally, the current mechanistic insights majorly rely on traditional cell culture assays and rodent models, while the limited utilization of advanced human organoid systems and targeted cell mechanistic validations continues to slow down the identification of their molecular targets and intracellular receptors interacting with *Lilium* metabolites.

Currently, the official international commercial footprint of the genus is heavily anchored within East Asian frameworks, specifically the Chinese Pharmacopoeia (2025 Edition), which strictly recognizes only three medicinal species, including *L. lancifolium*, *L. brownii* var. *viridulum*, and *L. pumilum* as official sources of Bai He [12]. The processed bulbs are incorporated into standardized pharmaceutical formulations, such as the Baihe Dihuang decoction, which are used for treating insomnia and perimenopausal emotional distress, historically termed Bai He disease [61,62].

Regardless of its translational limitations, the global patent landscape reflects rising commercial attention, with more than a hundred patents containing *Lilium*-related formulations. This commercial development has increasingly introduced non-pharmacopeial and highly vulnerable species into commercial supply chains, including *L. polyphyllum* (Kshira Kakoli) as an important therapeutic ingredient in high-value South Asian Ayurvedic rejuvenating tonics like Chyawanprash [63]. The pharmacological advancement of *Lilium* species is primarily limited by the clinical gap between in vivo preclinical animal studies and human validation. Despite extensive patent activities and preclinical in vivo data demonstrating anti-inflammatory, anti-aging, and anti-fibrotic effects, robust human clinical data remain limited. To date, few clinical studies have reported, like a 12-week human trial using *L. lancifolium* ethanol extract (1000 mg/d; oral dose) that confirmed the significant declines in Visual Analog Scale (VAS) joint pain, TNF-α, and IL-6 in arthritic adult patients [64]. However, most of the recent commercial therapeutic claims continue to depend solely on retrospective ethnobotanical reports and historical observation rather than prospective, double-blind randomized clinical trials (RCTs).

The reason for translational hindrance may directly be linked to the absence of biopharmaceutical data. While in silico computational methods like SwissADMET have provided initial reports to evaluate the pharmacokinetic potentials of selected fractions of *Lilium* species, but, still, these predictions remain inadequate for pharmaceutical advancement [13]. It has been noted that high-molecular-weight steroidal saponins, like lilioside, in general, show very low oral bioavailability. Their large, amphiphilic chemical structures may result in poor apparent permeability across intestinal epithelial layers, in addition to factors like gastrointestinal and metabolic biotransformation, which may affect the bioavailability and bioactivities of the compound in vivo. Transitioning the bioactive phytochemicals of *Lilium*, particularly its chief constituents like steroidal saponins and phenylpropanoid glycosides, into practical clinical drug products demands an in-depth analysis through the latest pharmacokinetic models.

To successfully transform *Lilium* phytochemicals into drug-like clinical molecules, modern research must shift away from raw administration toward cutting-edge pharmaceutical engineering and rational drug design. For example, encapsulation of *Lilium*-derived unstable or poorly permeable bioactive moieties within engineered polymeric nanoparticles, solid lipid nanoparticles (SLNs), or liposomes offers an effective method to increase their stability, bioavailability, avoid premature gastrointestinal degradation and increase target-tissue bioavailability. Similarly, structural modifications of the core *Lilium* saponin structure represent an opportunity to enhance their therapeutic potential and drug-like characteristics. Also, optimization of the specific functional group can decrease toxicity risks and improve lipophilicity to satisfy Lipinski’s Rule of Five, aligning these natural scaffolds with modern industrial drug design requirements.

Although historical evidence presents safe use of genus *Lilium* in traditional medicine and food in humans, with therapeutic dose ranges established at 6–12 g/day [12]. Though there are some contradictions regarding the toxicological profile of the genus *Lilium*, which demonstrate the need for a thorough systemic evaluation. Some *Lilium* species, like *L. tigrinum* or *L. longiflorum*, in veterinary medicine produce an acute toxicity even on minor quantities when ingested accidentally and cause rapid, severe epithelial necrosis of the renal tubules followed by fatal acute kidney injury (AKI) within 48–72 h [65,66].

Therefore, there is a need for public awareness and deeper comparative toxicological research to identify the specialized metabolic pathway producing this dramatic interspecies variation. In addition, a major gap in the existing toxicological studies is their reliance on acute toxicity studies, and there remains a significant lack of studies focusing on chronic and sub-chronic toxicity assessment for *Lilium* species. This limitation precludes long-term safety consideration and hampers the regulatory development of *Lilium*-based natural therapeutics. Future studies should prioritize comprehensive dose-toxicity measurements to evaluate potential organ-specific toxicity as well as cumulative adverse effects and safe therapeutic exposure limits.

Whereas the diverse *Lilium* species share broad genetic classifications, they show vast differences in regional adaptation and chemical potency. Among these, *Lilium polyphyllum* D. Don represents a distinctive species, which is restricted to the high-altitude alpine microclimate and presents a varied therapeutic profile and phytochemical metabolites that demand isolated, rigorous scientific evaluation.

## 7. *Lilium polyphyllum* D. Don ex Royle

*Lilium polyphyllum* is an important medicinal herb native to the Himalayan region and is used to treat a variety of illnesses in the traditional system of medicine in the subcontinent [16]. The plant is bulbous, which mostly attains a height of almost one meter. The leaves are lanceolate, slender, alternating, sessile and dazzling. The capsules have three angles and winged seeds, and the flowers are white with purple spots [16]. An underground stem with soft scales, leaves and a root connected by a basal plate and one or more growth points is called a bulb. Scales are the modified leaves that store food for the development of plant growth and supply nutrients to the growing plant until it has enough root system and is fully matured. The bulb peel reveals that cells have colors and a nucleus. *L. polyphyllum* has two kinds of roots: contractile and basal. The roots are crucial when growth begins in early spring and help in the absorption of nutrients and water, whereas the contractile roots protect the bulbs from frost damage during severe weather by anchoring and drawing the bulb deeper into the soil [67].

### 7.1. Distribution and Traditional Uses

**Figure 4:**
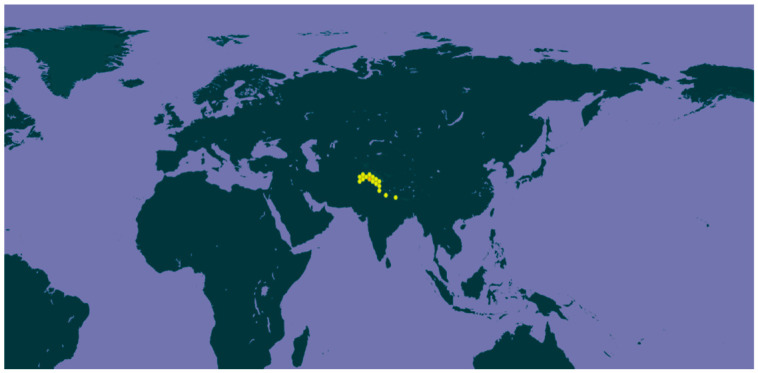
Global distribution of *Lilium polyphyllum*. Yellow circles indicate 31 unique georeferenced occurrence localities retained from an initial set of 163 GBIF records. Records lacking coordinates and duplicate coordinate pairs were excluded, and the remaining coordinates were checked for geographic plausibility. The map was generated using [software and version] in the WGS 84 coordinate reference system. Data source: GBIF.org, GBIF Occurrence Download, https://www.gbif.org/species/2753041 (accessed on 25 January 2026).

The plant *L. polyphyllum* is basically indigenous to the Himalayan areas, mainly between Uttarakhand (a state in India) and Afghanistan. It is also found in the USA, Canada and China [18]. Its global distribution is shown in Figure 4. In Pakistan, the plant is mainly distributed in Chitral, Kurram Valley, Swat, Upper Dir (Kohistan), Hazara and Murree [13]. In the Ayurvedic medical system, it is included under the Astravarga plants. Astavarga is a set of eight medicinal herbs that have revitalizing properties and are used to make a variety of Ayurvedic tonics [68].

The plant is used to make several Ayurvedic tonics, including Jivaniya ghrita, Astvarga Churan, and Chyawanprash. Because of its many medicinal uses, it is being overused to the point where it is considered a threatened plant species [69]. The plant *L. polyphyllum* has been used in traditional medicine to treat intermittent fever and sexual abnormalities. It has also been reported that the bulbs of *L. polyphyllum* are eaten in the Gangatori region of India. When fried in vegetable oil with potatoes, they may increase sexual arousal and serve as a source of warmth in the cold [70]. The plant bulbs have been noted for possessing anti-inflammatory, astringent, expectorant, aphrodisiac and diuretic properties. The bulbs are also used in Chyawanprash, an old-fashioned herbal remedy used in Ayurveda and in revitalizing night cream. Raw bulbs are consumed at high elevations to combat the cold [71].

### 7.2. Phytochemicals of Lilium polyphyllum

Phytochemical investigation of methanol/water extracts from dried roots using HPLC and GC-MS identified 26 compounds, including R-(-)-cyclohexylethylamine, longipinanol and macdougallin [18]. The GC-MS analysis of ethanol extract identified compounds like methyl 2-furoate, 5-hydroxymethyl furfural, methyl piperate, piperine, 7, 10-hexadecadienoic acid methyl ester, palmitic acid and methyl palmitate [72].

The GC-MS analysis of the methanolic extract, chloroform and n-hexane fractions of the bulbs identified different compounds such as heneicosane, eicosanoic acid methyl ester, 1-heptatriacotanol, DL-Pantolactone, divinyl sulfide, vanillin and 9-octadecenoic acid methyl ester, palmitic acid, methyl ester, linoleic acid, methyl ester, and myristic acid [13,73]. Similarly, the LC-TOP-MS/MS analysis of the ethyl acetate fraction identified several bioactive compounds, including 5′-O-acetyladenosine, ferulic acid methyl ester, kaempferol, hyperin and kaurenoic acid [13]. The details of the compounds identified, along with their structure and chemical classes, are given in Table 3.

### 7.3. Pharmacological Activities of Lilium polyphyllum

**Figure 5:**
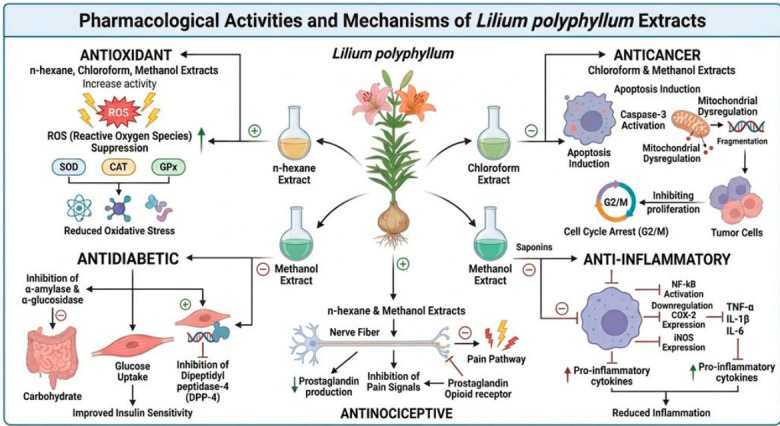
Schematic overview of the antioxidant, anticancer, anti-inflammatory, and antinociceptive activities of *Lilium polyphyllum* compiled from the reported literature cited in Section 7.3 and Table 4. Antioxidant activity is demonstrated by in vitro DPPH, ABTS, and FRAP assays, with the n-hexane fraction showing the highest potency. Anticancer effects are observed as cytotoxicity against HL-60 cells, particularly with chloroform and n-hexane fractions via apoptosis induction. Anti-inflammatory activity includes membrane stabilization, inhibition of oxidative burst, and reduction in carrageenan-induced paw edema in vivo. Antinociceptive effects are evidenced by decreased writhing responses and increased latency in hot plate tests, indicating both peripheral and central analgesic actions. Overall, the mechanisms shown are proposed, while the bioactivities are primarily based on preliminary in vitro and in vivo studies.

Previous studies have reported that *L. polyphyllum* exhibits diverse pharmacological potential (Figure 5). These include antioxidant, apoptotic, antiplatelet, antibacterial, antifungal, wound healing, anti-inflammatory, anti-nociceptive and antidiabetic effects as discussed in the following section and summarized in Table 4.

#### 7.3.1. Antioxidant Activity

Free radical-induced oxidative stress is primarily associated with the pathogenesis and progression of many degenerative conditions, including cancer, cardiovascular dysfunction and aging. Plant-derived antioxidants function by neutralizing reactive oxygen species (ROS) and mitigating oxidative damage in biological systems. Consequently, these phytoconstituents have emerged as focal points of intensive research within the pharmaceutical, cosmeceutical, and food industries due to their prophylactic and therapeutic potential [74,75].

Scientific evidence for the antioxidant potential of *L. polyphyllum* is limited but supported by in vitro assays. Methanolic extracts and their fractions (aqueous, n-hexane, ethyl acetate) have demonstrated free radical scavenging activity using DPPH, ABTS, and FRAP assays. Among the tested fractions, the n-hexane fraction exhibited the lowest IC_50_ (109.30 μg/mL), indicating the highest antioxidant potency, while aqueous and crude methanolic extracts also showed strong inhibition (>80% at higher concentrations). These findings suggest that non-polar constituents may significantly contribute to antioxidant effects. However, the absence of mechanistic studies and in vivo validation limits definitive conclusions.

Although the antioxidant activity of *L. polyphyllum* has been analyzed through in vitro assays including ABTS, FRAP and DPPH, these investigations basically evaluate free radical scavenging potential and do not evaluate biological antioxidant potential in living organisms. Presently, mechanistic studies involving cellular ROS modulation, Nrf2/HO-1 signaling cascades or in vivo oxidative stress markers remain lacking; hence, the reported antioxidant potential of this plant should be evaluated with caution, and further mechanistic evidence is needed to establish its biological relevance and pharmacological potential.

#### 7.3.2. Anticancer Activity

Cancer is one of the life-threatening diseases characterized by unregulated cellular proliferation and the evasion of programmed cell death, which remains a primary global health challenge [76,77]. Plant-derived natural products and their secondary metabolites, such as alkaloids, terpenoids, and flavonoids, possess significant anticancer potential by modulating key intracellular signaling pathways, including the induction of apoptosis and the inhibition of metastatic progression [78,79].

Preliminary studies indicate cytotoxic effects of *L. polyphyllum* bulb extracts. The n-hexane fraction demonstrated cytotoxicity against HL-60 leukemia cells (IC_50_ = 6.41 ± 0.20 μM), while the chloroform fraction showed even stronger activity (IC_50_ = 2.85 ± 0.16 μM), primarily through apoptosis induction and partial necrosis. Identified fatty acids such as hexadecanoic acid, linoleic acid, and tetradecanoic acid have been associated with anticancer effects in other studies [80,81]. Additionally, compounds like veridiflorol exhibit cytotoxic properties [82]. The current literature is primarily limited to cell viability and cytotoxic assays, while critical markers like selectivity toward tumor cells, migration and invasion inhibition, cell cycle arrest and molecular signaling mechanisms remain limitedly investigated. Additionally, the lack of an in vivo cancer model and cytotoxicity analysis limits the translational importance of these studies; hence, further preclinical and mechanism-based evaluation is needed to demonstrate the cytotoxic potential of this plant.

#### 7.3.3. Anti-Inflammatory Activity

The prevalence of inflammatory diseases is rising globally, particularly within aging populations. However, the clinical efficacy of available anti-inflammatory agents is often constrained by side effects and high economic costs. Consequently, natural products and traditional medicines have emerged as compelling alternatives, offering a diverse array of bioactive scaffolds with the potential for enhanced safety profiles and more cost-effective anti-inflammatory therapeutic interventions [83,84].

Both in vitro and in vivo studies support the anti-inflammatory potential of *L. polyphyllum*. In vitro, leaf extracts (especially aqueous) showed strong membrane stabilization activity (up to 88.81%), indicating inhibition of inflammatory processes. In vivo, methanolic extracts and their fractions significantly reduced carrageenan-induced paw edema at doses of 50–100 mg/kg. Furthermore, fractions such as chloroform, ethyl acetate, and n-hexane inhibited oxidative burst in whole blood assays, suggesting suppression of reactive oxygen species (ROS). Phytochemicals like diosgenin, kaempferol, berberine, and astragalin may contribute to these effects, although specific pathways remain unclear.

The anti-inflammatory potential of *L. polyphyllum* has been evaluated via preliminary studies such as carrageenan-induced paw edema in mice, membrane stabilization effect and oxidative burst inhibition assays, but these results provide limited mechanistic pathways into the underlying molecular processes. Currently, direct evidence about the modulation of principal inflammatory moieties and signaling pathways, including iNOS, COX-2, (TNF-α), (IL-6), (IL-1β), and MAPK pathways, remains insufficient, so the reported anti-inflammatory potential of *L. polyphyllum* should be further evaluated through advanced mechanistic in vivo investigations.

#### 7.3.4. Antinociceptive Activity

Pain is a complex sensory and emotional experience often managed by plant-derived extracts that exert antinociceptive effects by inhibiting the transmission of pain signals through central or peripheral pathways [85,86]. Numerous studies indicate that bioactive compounds such as alkaloids, flavonoids, and terpenoids can modulate pain pathways either via opioid receptors or inhibit inflammatory mediators like prostaglandins to reduce pain perception [87,88].

The antinociceptive potential of *L. polyphyllum* has been studied in animal models. The ethyl acetate fraction showed the most potent peripheral analgesic effect in the acetic acid-induced writhing test, significantly reducing abdominal constrictions at 50–100 mg/kg. Central analgesic activity was observed in the hot plate test, where fractions increased latency time, indicating modulation of pain perception. In silico studies further suggest interactions with pain-related targets such as P2 × 7 receptors. Howover, the analgesic effects of *L.polyphyllum* represent preliminary pharmacological evidence and do not elucidate the mechanistic pathways. Currently, no literature is available that investigates the involvement of opioid receptors using antagonists, the modulation of prostaglandin production or the regulation of pro-inflammatory cytokines and pain-related pathways; hence, mechanistic studies of the analgesic effect of this plant should be further evaluated via precise anti-nociceptive mechanisms.

**Table 4:** Pharmacological activities of *Lilium polyphyllum*.

Compound/Extract	Source	Pharmacological Activity	Disease/Model/Cell Line/Microorganism	In Vitro/In Vivo/In Silico	Treatment Concentration/IC_50_/Dose	Proposed Mechanism of Action	Key Effects/Findings	Reference
n-Hexane fraction (n-Hex-fr)	Bulb	Anticancer/ Cytotoxic	HL-60 leukemia cells	In vitro	IC_50_ = 6.41 ± 0.20 μM	Apoptosis induction (early and late stages)	Reduced cell viability; anti-proliferative effect	[73]
Chloroform fraction (Chl-fr)					IC_50_ = 2.85 ± 0.16 μM	Apoptosis induction; partial necrosis	Strong cytotoxicity; apoptosis induction	
Hydromethanolic extract (Cr. MeOH-Ext)					Not active	Not reported	No cytotoxic effect	
Aqueous fraction (Aq-fr)	Bulb	Antidiabetic (α-glucosidase inhibition)	Enzyme assay	In vitro	IC_50_ = 143.00 μg/mL	Enzyme inhibition	High % inhibition	
Cr. MeOH-Ext					IC_50_ = 203.30 μg/mL		Strong inhibition	
n-Hex-fr					IC_50_ = 174.60 μg/mL		Significant inhibition	
Chl-fr					IC_50_ = 358.70 μg/mL		Moderate inhibition	
EtOAc-fr					IC_50_ = 391.20 μg/mL		Moderate inhibition	
BuOH-fr					IC_50_ = 416.60 μg/mL		Moderate inhibition	
Cr. MeOH-Ext	Bulb	Antidiabetic (α-amylase inhibition)	Enzyme assay	In vitro	IC_50_ = 243.70 μg/mL	Enzyme inhibition	High inhibition	
Aq-fr					IC_50_ = 250.60 μg/mL		Highest inhibition	
Chl-fr					IC_50_ = 308.70 μg/mL		Moderate inhibition	
EtOAc-fr					IC_50_ = 316.90 μg/mL		Moderate inhibition	
BuOH-fr					IC_50_ = 456.70 μg/mL		Lower inhibition	
n-Hex-fr					IC_50_ = 692.00 μg/mL		Lowest inhibition	
Aq-fr	Bulb	Antioxidant (DPPH)	Radical scavenging assay	In vitro	IC_50_ = 184.10 μg/mL	Free radical scavenging	High antioxidant activity	
Cr. MeOH-Ext					IC_50_ = 687.40 μg/mL		Strong % inhibition	
n-Hex-fr					IC_50_ = 109.30 μg/mL		Lowest IC_50_ (highest potency)	
EtOAc-fr					IC_50_ = 182.30 μg/mL		Moderate activity	
Aq-fr					IC_50_ = 467.20 μg/mL		Strong inhibition	
n-Hex-fr					IC_50_ = 134.60 μg/mL		Highest activity	
*L. polyphyllum* extracts (water, ethanol, DCM)	Not specified	Antidiabetic α-amylase & α-glucosidase enzymes	Enzyme inhibition	In vitro	Amylase inhibition: water 54.95%, ethanol 53.01%, DCM 47.87%	Enzyme inhibition	Reduced carbohydrate breakdown	[89]
*L. polyphyllum* extracts (water, ethanol, DCM)					Water 48.48%, ethanol 48.48%, DCM 43.85%		Reduced glucose absorption	
Water extract	Leaf	Anti-inflammatory	HRBC membrane stabilization assay	In vitro	88.81% stabilization at 2.5 μg/mL	Membrane stabilization (anti-inflammatory)	Strong anti-inflammatory effect	[72]
Ethanolic extract					76.73% stabilization at 2.5 μg/mL		Moderate anti-inflammatory effect	
DCM extract					71.85% stabilization at 2.5 μg/mL		Lower activity	
Major phytochemicals (e.g., palmitic acid, FAMEs)	Bulb	ADMET/Toxicity prediction	Computational models	In silico	LD_50_ range: 775–20,000 mg/kg	Drug-likeness; low toxicity prediction	Favorable safety profile	[73]
Cr. MeOH-Ext (crude methanolic extract)	Bulb	Anti-inflammatory	Carrageenan-induced paw edema (animal model)	In vivo In vivo In vivo In vivo	50 and 100 mg/kg	Anti-inflammatory modulation (exact pathway not reported)	Significant reduction in paw edema	[13]
n-Hexane fraction (n-Hex-fr)			Carrageenan-induced paw edema		100 mg/kg	Anti-inflammatory modulation	Significant decrease in paw edema	
Chloroform fraction (Chl-fr)			Carrageenan-induced paw edema		100 mg/kg	Anti-inflammatory modulation	Significant reduction in inflammation	
Ethyl acetate fraction (EtOAc-fr)			Carrageenan-induced paw edema		100 mg/kg	Anti-inflammatory modulation	Significant reduction in inflammation	
Chl-fr, EtOAc-fr, n-Hex-fr			Whole blood oxidative burst assay	In vitro	Not reported	Inhibition of oxidative burst (ROS suppression)	Significant inhibition of inflammatory oxidative response	
EtOAc-fr	Bulb	Analgesic/ Antinociceptive	Acetic acid-induced writhing (animal model)	In vivo	50 and 100 mg/kg	Peripheral analgesic activity (not mechanistically detailed)	Significant reduction in abdominal writhing	
n-Hex-fr			Acetic acid writhing test		Not reported	Not reported	Reduced writhing (less effective than EtOAc-fr)	
Cr. MeOH-Ext			Acetic acid writhing test		Not reported	Not reported	Reduced writhing	
Chl-fr			Acetic acid writhing test		Not reported	Not reported	Reduced writhing	
EtOAc-fr			Hot plate test		100 mg/kg	Central analgesic activity (nociception modulation)	Increased latency time (pain threshold)	
Chl-fr			Hot plate test		Not reported	Not reported	Increased latency time	
n-Hex-fr			Hot plate test		Not reported	Not reported	Increased latency time	
Identified phytochemicals (mixed compounds)		Anti-inflammatory/Analgesic (target-based)	MPO and P2 × 7 receptor (protein targets)	In silico	Not reported	Strong binding to MPO and P2 × 7R (inflammation & pain pathways)	Supports anti-inflammatory and antinociceptive activity	
Cr. MeOH-Ext and fractions		Toxicity/Safety evaluation	Fibroblast cells, brine shrimp, animal model	In vitro + In vivo + In silico	Up to 2400 mg/kg (no toxicity)	Not reported	Non-toxic; favorable safety profile	

## 8. Conclusions and Future Perspectives

At present, the collective ethnomedicinal, phytochemical and pharmacological evidence highlights genus *Lilium* and *L. polyphyllum* as strategically significant with important potential for natural product-based drug discovery. The presence of diverse phenolic contents, steroidal saponins, polysaccharides and flavonoids, coupled with their broad spectrum of bioactivities and ethno-medicinal uses, provides a strong foundation for therapeutic development, but evaluation of their complete pharmacological potential needs mechanistic validation, standardization, pharmacokinetic validation and clinical evaluation.

The primary limitation of the existing *Lilium* literature is its overreliance on crude in vitro screenings without corresponding in vivo mechanistic studies and pharmacokinetic validation. Important data regarding human Absorption, Distribution, Metabolism, and Excretion (ADME) pathways are mostly limited. Addressing these gaps will be crucial for the successful conversion of *Lilium*-based metabolites into evidence-based pharmaceutical formulations. Furthermore, *L. polyphyllum* has become a highly susceptible species due to anthropogenic habitat degradation and unsustainable wild harvesting in its natural Himalayan habitat, threatening the survival of the resource itself. Addressing the current translation barriers and conserving measures of the important plants of *Lilium* and especially *L. polyphyllum* will necessitate the adoption of sustainable technologies, such as biotechnology, precision cultivation, bioactive compound optimization and product standardization.

Future advancement of *Lilium*-based pharmaceutical products needs biotechnological-based formulation systems, such as somatic embryogenesis, plant tissue culture, bio creator-based cultivation and metabolic engineering, which have the potential to provide standardized and pure quality phytochemicals while decreasing dependence on the wild population. These approaches should be incorporated with ex situ cultivation and domestication initiatives to provide a sustainable framework to reduce pressure on wild populations and pharmaceutical exploitation of endangered plant species. Similarly, advanced pharmaceutical development and nanotechnology-based carriers like polymeric nanoparticles, liposomes, solid lipid nanoparticles (SLNs), facilitate targeted drug delivery, stability, bioavailability and pharmacokinetic performance of *Lilium*-derived therapeutics.

Consequently, the future development of *Lilium*-based pharmaceuticals will rely on the traditional ethnopharmacological knowledge with modern-day biotechnological, advanced analytical techniques, pharmacological, mechanistic and clinical innovations. Similarly, conservation-based cultivation and biotechnology-driven production should be prioritized to protect the threatened genetic population. By coordinating these scientific advancements with active conservation and sustainable cultivation practices, the scientific community can fully realize the pharmaceutical potential of the genus *Lilium* while permanently protecting its endangered species from ecological extinction.

## Figures and Tables

**Table 2:** Bioactivities of *Lilium* species.

Plant Species	Part Used	Pharmacological Activity	Extract Type/Purified Compound	Disease/Model/Cell Line/Microorganism	Assay Type	Proposed Mechanism	References
*L. candidum*	Bulb	Anticancer	Methanolic	Human breast carcinoma cell line MCF-7 cells	In vitro	p53-mediated stimulation of apoptosis	[35]
*L. callosum*	Bulb		Callosum A	Cytotoxic activity against human cancer cell lines SGC-7901 (gastric carcinoma), K562 (human leukemia), and SPCA-1 (human lung adenocarcinoma)	In vitro	-	[36]
*L. brownii*	Root		Polysaccharide	S180 sarcoma-bearing mice model (murine tumor model)	In vivo	The water-soluble polysaccharides enhance immune function, promote cytokine production, stimulate immune cell function, and increase thymus and spleen indices, resulting in the inhibition of tumor cells.	[37]
*L. lancifolium*	Bulb		Butanol	A549 and H1299, human lung cancer cell lines	In vitro	Apoptosis induction and suppression of metastasis, linked with modulation of apoptosis-associated pathways	[38]
*L. lancifolium*	Leaves	Antioxidant	Polysaccharide	Antioxidant assay	In vitro	Direct free radical/ROS scavenging activity	[39]
*L. brownii*	Bulb		Polysaccharide	Chemical antioxidant assay	In vitro	Free radical scavenging and neutralization of ROS species	[40]
*L. davidii*	Root		Polysaccharide	Chemical antioxidant assay	In vitro	Free radical scavenging and neutralization of ROS species	[41]
*L. davidii*	Bulb		Polysaccharide	Antioxidant assay	In vitro	Free radical scavenging	[42]
*L. longiflorum*	Flowers		Crude extract	Antioxidant activity	In vitro	Free radical scavenging and cytoprotection	[43]
*L. candidum*	Bulb		1,2-O-diferuloylglycrrol	Chemical antioxidant assay	In vitro	Free radical scavenging, prevents oxidative damage via phenypropenoid glycerides	[44]
*L. lancifolium*	Bulb	Anti-inflammatory	Ethanolic	RAW 264.7 murine macrophage cells stimulated with lipopolysaccharide (LPS)	In vitro	Decrease NO, COX-2, iNOS, pro-inflammatory cytokines level	[45]
*L. brownii* *L. longiflorum* *L. pumilum*	Bulb		Methanolic	Inflammation/oxidative stress; mouse models	In vivo + In vitro	Suppression of inflammatory mediators	[46]
*L. lancifolium*	Roots		Aqueous	COPD-like pulmonary inflammation cigarette smoke-exposed mice	In vivo	Suppression of inflammatory mediators, protection against emphysematous lung injury	[47]
*L. lancifolium* *L. brownii* *L. pumilum*	Bulb		Aqueous	CCl_4_-induced liver fibrosis mouse model	In vivo	Inhibit TGF-β1/Smad2/3 signaling (anti-fibrotic effect), reduce inflammation, hepatoprotective	[48]
*L. brownii*	Bulb	Anti-depressant	Ethanolic	MPTP-induced Parkinson’s disease mouse model	In vivo	Neuroprotection through p62–Keap1–Nrf2-mediated antioxidant signaling, reduction in oxidative stress (↓ MDA, ↓ Fe^2+^, ↑ SOD, ↑ GSH-Px), protection of substantia nigra neurons, and improvement of motor deficits.	[49]
*L. lancifolium*	Bulb		Aqueous	Ovariectomized (OVX) mouse model	In vivo	Modulation of the brain–uterus axis and neuroendocrine pathways, improving behavioral symptoms without modulation as a conventional estrogen replacement therapy.	[50]
*L. brownii*	-		Saline	Stress-exposed BALB/c mice	In vivo	Increase hippocampal BDNF expression, decrease plasma corticosterone level	[51]
*L. lancifolium Thunb*	Bulb		Ethanolic	Chronic mild stress (CMS/CUMS) rat model	In vivo	Activation of BDNF/TrkB signaling pathway, regulates neuroinflammation, and monoamine neurotransmitters	[52]
*L. davidii*	Bulb	Anti-bacterial	Polysaccharide	*E. coli*, *S. aureus*, *B. subtilis*	In vitro	Inhibit bacterial growth, DPPH, hydroxyl and superoxide radical scavenging	[42]
*L. lancifolium* *L. brownii* *L. pumilum*	Root		Colchicine	-	*-*	-	[1]
*L. lancifolium*	Bulb		Aqueous	*E. coli*, *S. aureus*, *B. subtilis*	In vitro	Inhibit bacterial growth	[53]
*L.lancifolium*	Bulb		Ethanolic	*E. coli*, *S. aureus*, *B. subtilis*	In vitro	Inhibit bacterial growth	[53]
*L. lancifolium*	Bulb		Ethyl acetate	*E. coli*, *S. aureus*, *B. subtilis*	In vitro	Inhibit bacterial growth	[53]
*L. pumilum*	-	Hepato-protectant	Methanolic and Water	Isolated perfused rat liver	In vitro (Ex vivo)	Increased bile secretion and bile flow (choleretic effect) through stimulation of hepatocellular secretory function.	[54]
*L. longiflorum*	Bulb		Butanol and ethanol	CCl_4_-induced hepatotoxicity mouse model	In vivo	Hepatoprotection through antioxidant activity, reduction in ALT/AST levels	[55]
*L. brownii*	Bulb		Aqueous	CCl_4_-induced liver fibrosis mouse model	In vivo	Inhibit TGF-β1/Smad2/3 signaling (anti-fibrotic effect), reduce inflammation, hepatoprotective	[48]
*L. candidum*	Bulb	Sodium/potassium ATPase inhibition	Steroidal saponins (glycoside)	Na^+^/K^+^-ATPase enzyme inhibition assay	In vitro	Direct Na^+^/K^+^-ATPase inhibition by isolated steroidal saponins	[56]
*L. pumilum*	Bulb		Steroidal saponins (glycoside)	Na^+^/K^+^-ATPase enzyme inhibition assay	In vitro	Inhibition of Na^+^/K^+^-ATPase by steroidal and phenolic glycosides	[57]

**Table 3:** Phytochemical profile of *Lilium polyphyllum* [13,18,72,73].

Phytochemical	Chemical Class	Formula	Structure	Mode of Isolation/ Identification	Extract Type	Part Used
R-(-)-Cyclohexylethylamine	Chiral mine	C_8_H_17_N	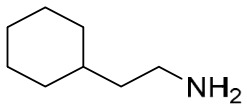	GC-MS	Chloroform and ethyl acetate fractions	Bulbs
Pregan-20-one,2-hydroxy-5,6-epoxy-15-methyl	Steroid	C_22_H_34_O_3_	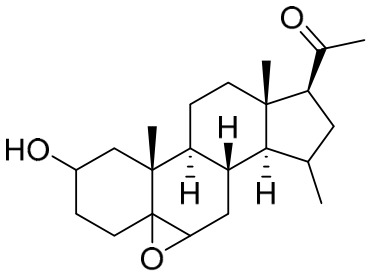	HPLC	(MeOH/H_2_O) (9:1)	Dried roots
1-(5-Bicyclo [2.2.1] heptyl) ethylamine	Chiral amine	C_9_H_17_N	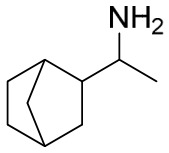	HPLC	(MeOH/H_2_O) (9:1)	Dried roots
1-(3,5-Dimethyl-1-adamantanoyl) semicarbazide	Adamantane derivative	C_14_H_23_N_3_O_2_	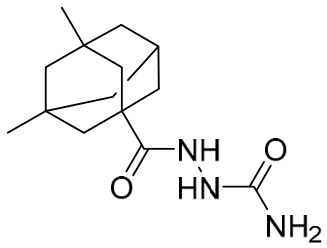	HPLC	(MeOH/H_2_O) (9:1)	Dried roots
Eudesmol	Oxygenated sesquiterpene	C_15_H_26_O	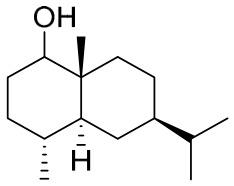	HPLC	(MeOH/H_2_O) (9:1)	Dried roots
(S)-(+)-1-Cyclohexylethylamine	Chiral amine	C_8_H_17_N	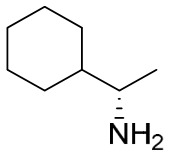	HPLC	(MeOH/H_2_O) (9:1)	Dried roots
1,3-Adamantanediacetamide	Adamantane derivative	C_14_H_22_N_2_O_2_	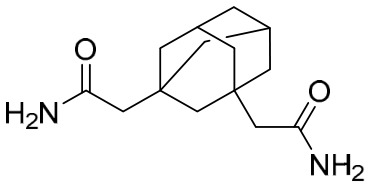	HPLC	(MeOH/H_2_O) (9:1))	Dried roots
Columbin	Diterpenoid-furanolactone	C_20_H_22_O_6_	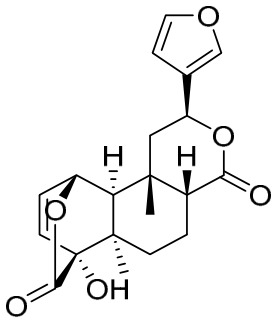	HPLC	(MeOH/H_2_O) (9:1)	Dried roots
Methyl 2-furoate	Acid	C_6_H_6_O_3_	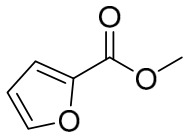	Column chromatography or preparative HPLC/HPLC, GC-MS	Ethanol extract	Whole plant
8α-Acetoxyelemol	Oxygenated sesquiterpene	C_17_H_28_O_3_	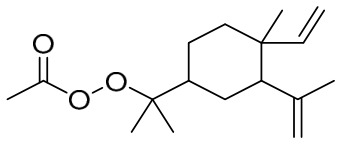	HPLC	(MeOH/H_2_O) (9:1)	Dried roots
Tris-(dimethylamino) methane	Cyclo-amino alkane	C_7_H_19_N_3_	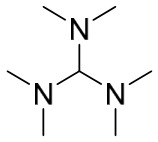	HPLC	(MeOH/H_2_O) (9:1)	Dried roots
α-Methyl-1-adamantanemethylamine	Adamantane derivative	C_12_H_21_N	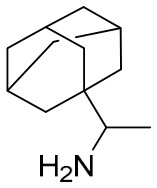	HPLC	(MeOH/H_2_O) (9:1)	Dried roots
Palustrol	Sesquiterpene	C_15_H_26_O	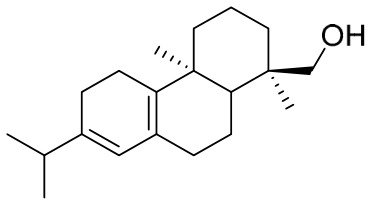	HPLC	(MeOH/H_2_O) (9:1)	Dried roots
7-Oxabicyclo [4.1.0] heptane, 1-methyl-4-(2-methyloxiranyl)	Oxygenated monoterpene	C_10_H_16_O_2_	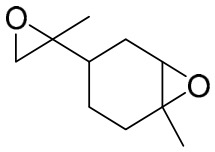	HPLC	(MeOH/H_2_O) (9:1)	Dried roots
5-hydroxymethyl furfural	Furan	C_6_H_6_O_3_	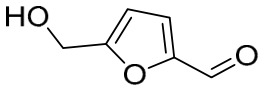	Column chromatography or preparative HPLC, GC-MS	Ethanol extract	Whole Plant
Palmitic acid	Acid	C_16_H_32_O_2_	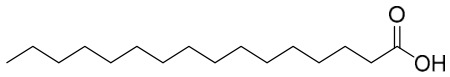	GC-MS	Cr. MeOH-Extract	Bulbs
Estran-3-one,17 (acetyloxy)-2-methyl, (2, 5 α, 5, 17 β)	Steroid	C_21_H_32_O_3_	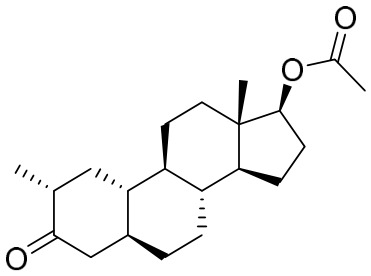	HPLC	(MeOH/H_2_O) (9:1)	Dried roots
cis-Z-α-Bisabolene epoxide	Oxygenated sesquiterpene	C_15_H_24_O	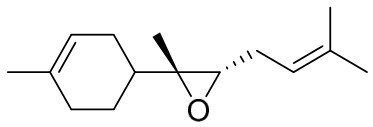	HPLC	(MeOH/H_2_O) (9:1)	Dried roots
1-Heptatriacotanol	Alcohol	C_37_H_76_O	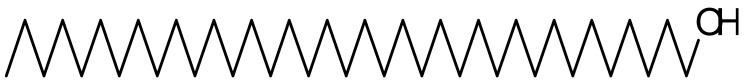	HPLC	(MeOH/H_2_O) (9:1)	Dried roots
Veridiflorol	Sesquiterpene alcohol	C_15_H_26_O	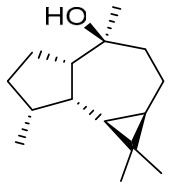	HPLC	(MeOH/H_2_O) (9:1)	Dried roots
Methyl piperate	Piperine alkaloid	C_13_H_12_O_4_	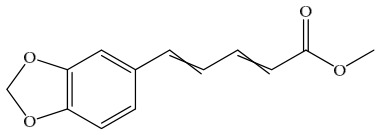	Column chromatography or preparative HPLC, GC-MS	Ethanol extract	Whole Plant
7,10-Hexadecadienoic acid methyl ester	Ester	C_17_H_30_O_2_	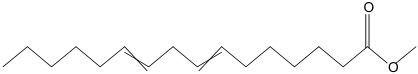	Column chromatography or preparative HPLC, GC-MS	Ethanol extract	Whole Plant
Piperine	Alkaloid	C_17_H_19_NO_3_	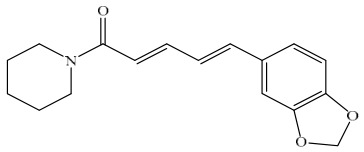	Column chromatography or preparative HPLC, GC-MS	Ethanol extract	Whole Plant
Heneicosane	Hydrocarbon	C_21_H_44_	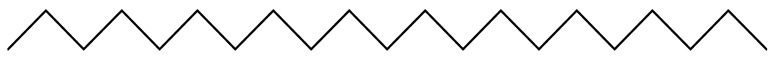	GC-MS	Cr. MeOH-Extract	Bulbs
Eicosanoic acid methyl ester,	Fatty acid	C_21_H_42_O_2_	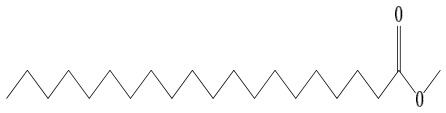	GC-MS	Cr. MeOH-Extract	Bulbs
1-heptatriacotanol	Fatty alcohol	C_37_H_76_O	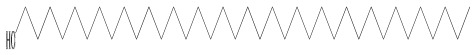	GC-MS	Cr. MeOH-Extract	Bulbs
Octane, 2,4,6-trimethyl	Hydrocarbon	C_11_H_24_	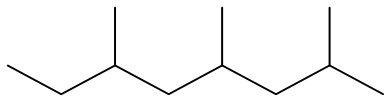	GC-MS	Cr. MeOH-Extract	Bulbs
Vanillin	Phenolic aldehyde	C_8_H_8_O_3_	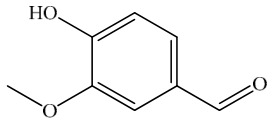	GC-MS	Chloroform fraction	Bulbs
9-octadecenoic acid methyl ester	Fatty acid methyl ester	C_19_H_34_O	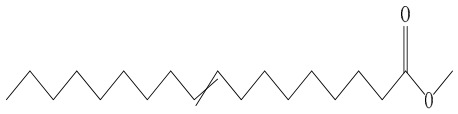	GC-MS	Chloroform fraction	Bulbs
Palmitic acid, methyl ester	Fatty acid	C_17_H_34_O_2_	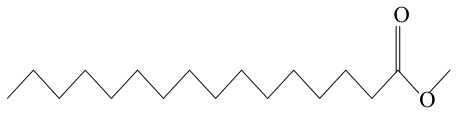	GC-MS	n-hexane fraction	Bulbs
Linoleic acid, methyl ester	Fatty acid	C_19_H_34_O_2_	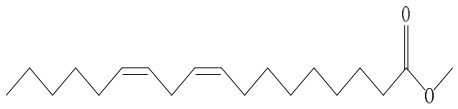	GC-MS	n-hexane fraction	Bulbs
Myristic acid	Fatty acid	C_17_H_28_O_2_	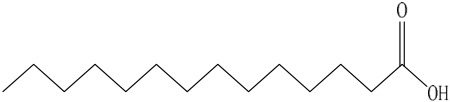	GC-MS	n-hexane fraction	Bulbs
5′-O-Acetyladenosine	Nucleoside	C_12_H_15_N_5_O_5_	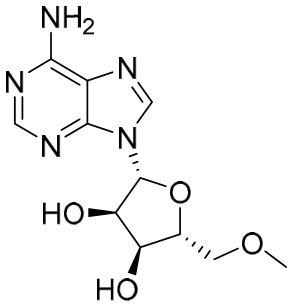	LC-QTOF-MS	Ethyl acetate fraction	Bulbs
Caffeic acid	Phenolic acid	C_9_H_8_O_4_	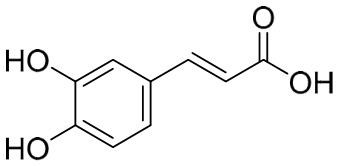	LC-QTOF-MS	Ethyl acetate fraction	Bulbs
Ferulic acid methyl ester	Phenolic ester	C_11_H_12_O_4_	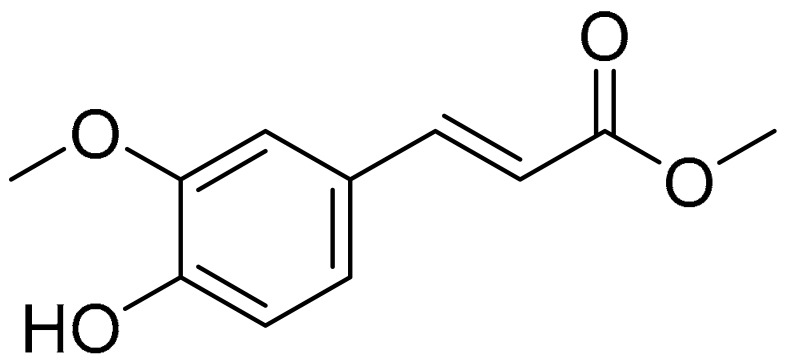	LC-QTOF-MS	Ethyl acetate fraction	Bulbs
Isophorone	Cyclic ketone	C_9_H_14_O	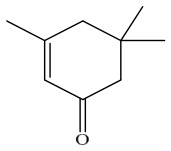	LC-QTOF-MS	Ethyl acetate fraction	Bulbs
Kaempferol	Flavonoid	C_15_H_10_O_6_	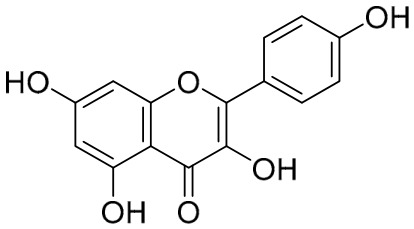	LC-QTOF-MS	Ethyl acetate fraction	Bulbs
Hyperin	Flavonoid glycoside	C_21_H_20_O_12_	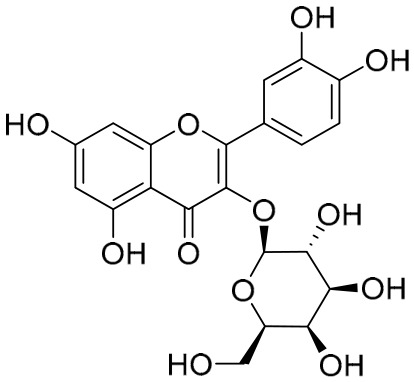	LC-QTOF-MS	Ethyl acetate fraction	Bulbs
Tigogenin	Steroidal sapogenin	C_27_H_44_O_3_	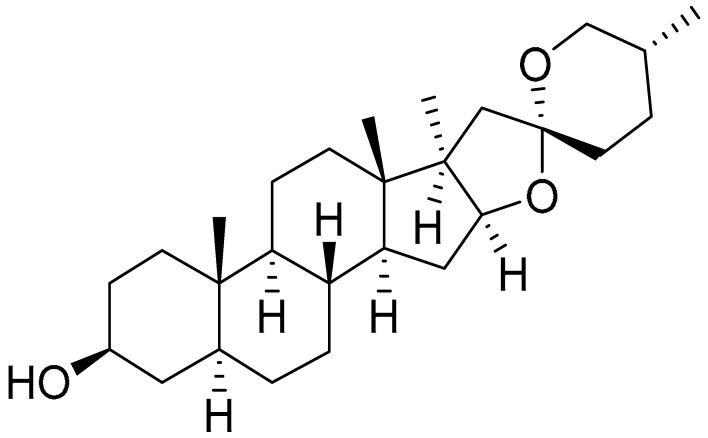	LC-QTOF-MS	Ethyl acetate fraction	Bulbs
Harmine	Indole alkaloids	C_13_H_12_N_2_O	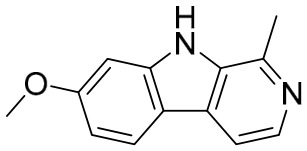	LC-QTOF-MS	Ethyl acetate fraction	Bulbs
Kaurenoic acid	Terpenoid	C_20_H_30_O_2_	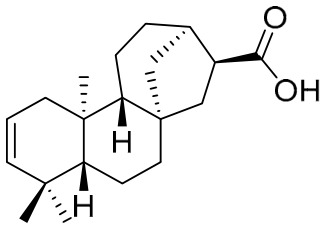	LC-QTOF-MS	Ethyl acetate fraction	Bulbs

## Data Availability

No new data were created or analyzed in this study. Data sharing is not applicable to this article.

## References

[1] Zhou J., An R., Huang X. (2021). Genus *Lilium*: A review on traditional uses, phytochemistry and pharmacology. J. Ethnopharmacol..

[2] Du Y.-P., He H.-B., Wang Z.-X., Wei C., Li S., Jia G.-X. (2014). Investigation and evaluation of the genus *Lilium* resources native to China. Genet. Resour. Crop Evol..

[3] Zhou P., Liao K., Feng X., Liang R., Teng N., Du F. (2025). Morphological and Anatomical Characterization of Stems in *Lilium* Taxa. Horticulturae.

[4] Van Tuyl J.M., Arens P., Shahin A., Marasek-Ciołakowska A., Barba-Gonzalez R., Kim H.T., Lim K.-B. (2018). *Lilium*. Ornamental Crops.

[5] Saifullah K., Sheeba N., Mariam R., Naheed K., Asma N., Bushra S. (2010). Cultivation of lilies (*Lilium regale*) for commercialization in Pakistan. Pak. J. Bot..

[6] Dhiman M., Moudgil S., Parkash C., Kumar R., Kumar S. (2018). Biodiversity in *Lilium*: A review. Int. J. Hortic..

[7] Yu P., He W., Hu J., Zhang B., Quan H., Lan X., Guo X. (2025). Phytochemical Profiles and Melanin Inhibition Potential of Three *Lilium* Species. ACS Agric. Sci. Technol..

[8] Lupșor S., Stanciu G., Cristache R.E., Pănuș E., Radulescu C., Olteanu R.L., Buruleanu C.L., Stirbescu R.M. (2025). Phytochemical evaluation and antioxidant-antimicrobial potential of *Lilium* spp. bulbs: Therapeutic and dermatocosmetic applications. Plants.

[9] Chatterjee S., Kumar D., Goswami P.K., Patel A.K. (2026). Medicinal Innovation Source of Liliaceae: Mapping Phytochemical Diversity and Pharmacological Breakthroughs. Curr. Tradit. Med..

[10] Wang Y.-F., An Z.-Y., Yuan L.-Q., Wang T., Jin W.-L. (2024). *Lilium brownii*/Baihe as nutraceuticals: Insights into its composition and therapeutic properties. Pharmaceuticals.

[11] Pan W., Shi H., Zang Z., Meng Q., Cheng Y., Liang L., Zhai Y., Yin G., Sun L., Ma K. (2024). Research progress on classical traditional Chinese medicine formula Baihe Zhimu (*Lilium lancifolium* bulb and *Anemarrhena asphodeloides* rhizome) decoction in the treatment of depression. Heliyon.

[12] Wang Y., Chen H., Feng P., Wang D., Du X. (2025). Traditional uses, nutritional properties, phytochemical metabolites, pharmacological properties, and potential applications of *Lilium* spp.: A systematic review. Front. Pharmacol..

[13] Ali H., Ur Rahman S., Sayad U., Khalil A.A.K., Abbas M., Shahid M. (2026). Phytochemical Profiling and Evaluation of the Anti-inflammatory and Antinociceptive Effects of *Lilium polyphyllum* Bulb Using in silico, in vitro and in vivo models. J. Pharm. Innov..

[14] Paul S., Samant S., Lal M. (2025). Population ecology and influence of climate change on the distribution of a critically endangered medicinal plant *Lilium polyphyllum* in the Himalaya. Proc. Indian Natl. Sci. Acad. USA.

[15] Dhyani A., Nautiyal B.P., Nautiyal M.C. (2022). Seedling performance, phenotypic traits and growing media for domestication of *Lilium polyphyllum*. Plants People Planet.

[16] Rana M.S., Samant S.S. (2011). Population biology of *Lilium polyphyllum* D. Don ex Royle a critically endangered medicinal plant in a protected area of Northwestern Himalaya. J. Nat. Conserv..

[17] Bisht A.S. (2020). *Lilium polyphyllum* D. Don Ex. Royle: A critically endangered medicinal plant of western himalaya. Plant Biodiversity and Bioactives.

[18] Raval S., Mandavia M., Mahatma M., Golakiya B. (2015). Separation and identification of phytochemicals from *Lilium polyphyllum* D. Don (kshirkakoli), an ingredient of Ashtavarga. J. Cell Tissue Res..

[19] Dhyani A., Nautiyal B.P., Yadav V., Nautiyal M.C. (2023). Variation in morphological, biochemical and antioxidant properties of *Lilium polyphyllum*. Indian J. Biochem. Biophys. (IJBB).

[20] Bera K., Ghosh P., Halder S., Naskar N., Bhowmik S., Mondal S., Bairagi A., Purkait A. (2022). A review on pharmacological and phytochemical activities of *Lilium polyphyllum* (Liliaceae): Himalaya lily. Int. J. Innov. Sci. Res. Technol..

[21] Dhyani A., Sharma G., Nautiyal B.P., Nautiyal M.C. (2014). Propagation and conservation of *Lilium polyphyllum* D. Don ex Royle. J. Appl. Res. Med. Aromat. Plants.

[22] Basit A., Lim K.-B. (2025). Recent approaches towards characterization, genetic, and genomic perspectives of genus *Lilium*. Genet. Resour. Crop Evol..

[23] Dhyani A., Nautiyal B.P., Nautiyal M.C. (2017). Distribution, status and conservation of *Lilium polyphyllum* (Liliaceae), a critically endangered medicinal plant from India. Plant Biosyst.-Int. J. Deal. All Asp. Plant Biol..

[24] Dhyani A., Baskin C.C., Nautiyal B.P., Nautiyal M.C. (2019). Overcoming root dormancy and identifying the storage behaviour of *Lilium polyphyllum* seeds. Botany.

[25] Kundra R., Samant S.S., Kumar V., Pande V. (2020). Callus mediated organogenesis in *Lilium polyphyllum* D. Don: A critically endangered Astavarga plant from Northwestern Indian Himalaya. Med. Plants-Int. J. Phytomed. Relat. Ind..

[26] Li J., Sun M., Li H., Ling Z., Wang D., Zhang J., Shi L. (2022). Full-length transcriptome-referenced analysis reveals crucial roles of hormone and wounding during induction of aerial bulbils in lily. BMC Plant Biol..

[27] Saadon S., Zaccai M. (2013). *Lilium candidum* bulblet and meristem development. Vitr. Cell. Dev. Biol.-Plant.

[28] eFloras.org *Lilium* in Flora of China. http://www.efloras.org/florataxon.aspx?flora_id=2&taxon_id=118558.

[29] eFloras.org Ornamental Plants from Russia. http://www.efloras.org/florataxon.aspx?flora_id=120&taxon_id=200027720.

[30] eFloras.org Flora of North America. http://www.efloras.org/florataxon.aspx?flora_id=1&taxon_id=242101743.

[31] Missouri Botanical Garden. https://www.missouribotanicalgarden.org/PlantFinder/PlantFinderDetails.aspx?taxonid=282318.

[32] eFloras.org Flora of Pakistan. http://www.efloras.org/florataxon.aspx?flora_id=5&taxon_id=250096012.

[33] Kalaba V., Sladojević Ž., Balaban Ž.M., Kalaba D., Panić I. (2019). Antibacterial properties of white lily (*Lilium candidum*) extract. Vet. J. Repub. Srp..

[34] Pragya Sourabh P.S., Julie Thakur J.T., Uniyal P., Pandey A. (2015). Biology of *Lilium polyphyllum*-A threatened medicinal plant. Int. J. Phytomed. Relat. Ind..

[35] Tokgun O., Akca H., Mammadov R., Aykurt C., Deniz G. (2012). *Convolvulus galaticus*, *Crocus antalyensis*, and *Lilium candidum* extracts show their antitumor activity through induction of p53-mediated apoptosis on human breast cancer cell line MCF-7 cells. J. Med. Food.

[36] Wang X., Wu G.Q. (2014). A new steroidal glycoside and potential anticancer cytotoxic activity of compounds isolated from the bulbs of *Lilium callosum*. J. Chem. Res..

[37] Sun X., Gao R.-L., Xiong Y.-K., Huang Q.-C., Xu M. (2014). Antitumor and immunomodulatory effects of a water-soluble polysaccharide from Lilii Bulbus in mice. Carbohydr. Polym..

[38] Luo L.-M., Qin L., Zhan J.-H., Pei G., Zhou X.-J., Chen N.-H. (2018). Study on effects of total saponins from Lilii Bulbus on proliferation, apoptosis, invasion and metastasis of lung cancer cells and its preliminary mechanism. China J. Chin. Mater. Medica.

[39] Xu Z., Wang H., Wang B., Fu L., Yuan M., Liu J., Zhou L., Ding C. (2016). Characterization and antioxidant activities of polysaccharides from the leaves of *Lilium lancifolium* Thunb. Int. J. Biol. Macromol..

[40] Gao J., Zhang T., Jin Z.-Y., Xu X.-M., Wang J.-H., Zha X.-Q., Chen H.-Q. (2015). Structural characterisation, physicochemical properties and antioxidant activity of polysaccharide from *Lilium lancifolium* Thunb. Food Chem..

[41] Hui H., Jin H., Li X., Yang X., Cui H., Xin A., Zhao R., Qin B. (2019). Purification, characterization and antioxidant activities of a polysaccharide from the roots of *Lilium davidii* var. *unicolor* Cotton. Int. J. Biol. Macromol..

[42] Hui H., Li X., Jin H., Yang X., Xin A., Zhao R., Qin B. (2019). Structural characterization, antioxidant and antibacterial activities of two heteropolysaccharides purified from the bulbs of *Lilium davidii* var. *unicolor* Cotton. Int. J. Biol. Macromol..

[43] Galova E., Kopaskova M., Sevcovicova A., Hadjo L., Yankulova B., Gregan F., Chankova S., Miadokova E. (2011). The role of antioxidants from *Lilium candidum* L. and *Salvia officinalis* L. Extracts in phytomedicine. Toxicol. Lett..

[44] Luo J., Li L., Kong L. (2012). Preparative separation of phenylpropenoid glycerides from the bulbs of *Lilium lancifolium* by high-speed counter-current chromatography and evaluation of their antioxidant activities. Food Chem..

[45] Sim W.S., Choi S.I., Jung T.D., Cho B.Y., Choi S.H., Park S.M., Lee O.H. (2020). Antioxidant and anti-inflammatory effects of *Lilium lancifolium* bulbs extract. J. Food Biochem..

[46] Wang T., Huang H., Zhang Y., Li X., Li H., Jiang Q., Gao W. (2015). Role of Effective Composition on Antioxidant, Anti-inflammatory, Sedative-Hypnotic Capacities of 6 Common Edible *Lilium* Varieties. J. Food Sci..

[47] Lee E., Yun N., Jang Y.P., Kim J. (2013). *Lilium lancifolium* Thunb. extract attenuates pulmonary inflammation and air space enlargement in a cigarette smoke-exposed mouse model. J. Ethnopharmacol..

[48] Chen Y., Li R., Hu N., Yu C., Song H., Li Y., Dai Y., Guo Z., Li M., Zheng Y. (2020). Baihe Wuyao decoction ameliorates CCl4-induced chronic liver injury and liver fibrosis in mice through blocking TGF-β1/Smad2/3 signaling, anti-inflammation and anti-oxidation effects. J. Ethnopharmacol..

[49] Hui C., Jin J., Ji M., Wang H., Wang X., Ma J., Wang Y., Si Y., Chen S., Guo T. (2024). Neuroprotective properties of the *Lilium brownii* extracts in the experimental model of Parkinson’s disease. Metab. Brain Dis..

[50] Zhou X.-D., Shi D.-D., Wang H.-N., Tan Q.-R., Zhang Z.-J. (2019). Aqueous extract of lily bulb ameliorates menopause-like behavior in ovariectomized mice with novel brain-uterus mechanisms distinct from estrogen therapy. Biomed. Pharmacother..

[51] Doron R., Lotan D., Rak-Rabl A., Raskin-Ramot A., Lavi K., Rehavi M. (2012). Anxiolytic effects of a novel herbal treatment in mice models of anxiety. Life Sci..

[52] Jia D., Dou Y., He Y., Zhou X., Gao Y., Ma M., Wu Z., Li W. (2020). Saponin extract of Baihe-Zhimu Tang ameliorates depression in chronic mild stress rats. J. Funct. Foods.

[53] Zhou Y., Duan Z., Wang H., Li C., Huang C. (2008). Study on antibacterial activities of extracts of *Lilium lancifolium* Thunb. Food Sci..

[54] Obmann A., Tsendayush D., Thalhammer T., Zehl M., Vo T.P.N., Purevsuren S., Natsagdorj D., Narantuya S., Kletter C., Glasl S. (2010). Extracts from the Mongolian traditional medicinal plants Dianthus versicolor Fisch. and *Lilium pumilum* Delile stimulates bile flow in an isolated perfused rat liver model. J. Ethnopharmacol..

[55] Tang W., Munafo J.P., Palatini K., Esposito D., Huang M.-T., Komarnytsky S., Ho C.-T., Gianfagna T.J. (2015). Hepatoprotective activity of easter lily (*Lilium longiflorum* Thunb.) bulb extracts. J. Agric. Food Chem..

[56] Mimaki Y., Satou T., Kuroda M., Sashida Y., Hatakeyama Y. (1999). Steroidal saponins from the bulbs of *Lilium candidum*. Phytochemistry.

[57] Zhou Z.-L., Feng Z.-C., Fu C.-Y., Zhang H.-L., Xia J.-M. (2012). Steroidal and phenolic glycosides from the bulbs of *Lilium pumilum* DC and their potential Na^+^/K^+^ ATPase inhibitory activity. Molecules.

[58] Yuan Z.Y., Li Z.Y., Zhao H.Q., Gao C., Xiao M.W., Jiang X.M., Zhu J.P., Huang H.Y., Xu G.M., Xie M.Z. (2021). Effects of different drying methods on the chemical constituents of *Lilium lancifolium* Thunb. based on UHPLC-MS analysis and antidepressant activity of the main chemical component regaloside A. J. Sep. Sci..

[59] Ma T., Wang Z., Zhang Y.-M., Luo J.-G., Kong L.-Y. (2017). Bioassay-guided isolation of anti-inflammatory components from the bulbs of *Lilium brownii* var. *viridulum* and identifying the underlying mechanism through acting on the NF-κB/MAPKs pathway. Molecules.

[60] Qi L., Qian S., Wang L., Liu Z., Jiang J., Liu C., Pan Y., Wang B., Han X., Chen J. (2026). Properties of Approved Antitumour Chinese Herbal Medicines: Integrating Evidence and Tradition. Drug Des. Dev. Ther..

[61] Peng L., Zhang X.F., Guo D.Y., Zhai B.T., Liang Y.J., Chen Z.Z., Zou J.B., Shi Y.J. (2022). Evaluation of the Clinical Efficacy of the Classic Prescription “Baihe Dihuang Decoction” Based on Meta-Analysis. Evid.-Based Complement. Altern. Med..

[62] Beebe S. (2023). Bai He Gu Jin Tang (Lily Bulb Decoction to Preserve the Metal): A Chinese Herbal Medication for the Metal Element. Am. J. Tradit. Chin. Vet. Med..

[63] Raval S., Mandavia M., Sanghani J., Mahatma M., Golakiya B. (2015). Separation and identification of phytochemicals from *Roscoea procera* Wall. (Kakoli), an ingredient of Ashtavarga. Indian J. Agric. Biochem..

[64] Jeon S., Lee H., Lee J.-H., Lee K., Hong D., Park S.-D., Shim J.-J., Lee J.-L., Lee J., Joo J.-C. (2024). The effects of *Lilium lancifolium* thunb. On the alleviation of joint pain: A randomized, double-blind, placebo-controlled clinical trial. Life.

[65] Fitzgerald K.T. (2010). Lily toxicity in the cat. Top. Companion Anim. Med..

[66] To A., Davila C., Stroope S., Walton R. (2023). Case report: Resolution of oligo-anuric acute kidney injury with furosemide administration in a cat following lily toxicity. Front. Vet. Sci..

[67] Dhyani A., Bahuguna Y.M., Semwal D.P., Nautiyal B.P., Nautiyal M.C. (2009). Anatomical features of *Lilium polyphyllum* D. Don ex Royle (Liliaceae). J. Am. Sci..

[68] Harriman N.A. (2004). Flora of Pakistan. Econ. Bot..

[69] Sahu R., Itankar P., Mishra R., Maliye A. (2016). Pharmacological evaluation of *Roscea procera* (Kakoli) and *Lilium polyphyllum* (Kshirkakoli) extracts for immunomodulatory activity. Int. J. Pharmacogn. Phytochem. Res..

[70] Li J.-W., Zhang X.-C., Wang M.-R., Bi W.-L., Faisal M., Da Silva J.A.T., Volk G.M., Wang Q.-C. (2019). Development, progress and future prospects in cryobiotechnology of *Lilium* spp.. Plant Methods.

[71] Panwar G., Srivastava S., Uniyal P. (2017). Callus-mediated organogenesis in *Lilium polyphyllum* D. Don ex Royle: A critically endangered Astavarga plant. Curr. Sci..

[72] Mir M.A., Ashraf M.W., Singh P. (2021). Phytochemical isolation and anti-inflammatory properties of various extracts of *Lilium polyphyllum*. Res. J. Pharm. Technol..

[73] Ali H., Rahman S.U., Rahman N., Zafar H., Sayad U., Khalil A.A.K. (2026). Gas Chromatography-Mass Spectrometry-Based Phytochemical Composition and Bioactivities of *Lilium polyphyllum* Bulb Extracts: Insights Into Anticancer and Antidiabetic Potential. Chem. Biodivers..

[74] Poljsak B., Šuput D., Milisav I. (2013). Achieving the balance between ROS and antioxidants: When to use the synthetic antioxidants. Oxidative Med. Cell. Longev..

[75] Khalil A.A.K., Bae H. (2025). Exploring the medicinal potential of *Alnus japonica*: A comprehensive review of phytochemicals and therapeutic applications. Naunyn-Schmiedeberg’s Arch. Pharmacol..

[76] Brierley J.D., Sullivan R. (2025). Union for international cancer control (UICC). Cancer Syst. Control Health Prof..

[77] Singh A., Roghini S. (2023). Cancer: Unraveling the complexities of uncontrolled growth and metastasis. PEXACY Int. J. Pharm. Sci..

[78] Sun L.-R., Zhou W., Zhang H.-M., Guo Q.-S., Yang W., Li B.-J., Sun Z.-H., Gao S.-H., Cui R.-J. (2019). Modulation of multiple signaling pathways of the plant-derived natural products in cancer. Front. Oncol..

[79] Gahtori R., Tripathi A.H., Kumari A., Negi N., Paliwal A., Tripathi P., Joshi P., Rai R.C., Upadhyay S.K. (2023). Anticancer plant-derivatives: Deciphering their oncopreventive and therapeutic potential in molecular terms. Future J. Pharm. Sci..

[80] Bharath B., Perinbam K., Devanesan S., AlSalhi M.S., Saravanan M. (2021). Evaluation of the anticancer potential of Hexadecanoic acid from brown algae *Turbinaria ornata* on HT–29 colon cancer cells. J. Mol. Struct..

[81] Mohan S., Bustamam A., Ibrahim S., Al-Zubairi A.S., Aspollah M., Abdullah R., Elhassan M.M. (2011). In Vitro Ultramorphological Assessment of Apoptosis on CEMss Induced by Linoleic Acid-Rich Fraction from *Typhonium flagelliforme* Tuber. Evid.-Based Complement. Altern. Med..

[82] Akiel M.A., Alshehri O.Y., Aljihani S.A., Almuaysib A., Bader A., Al-Asmari A.I., Alamri H.S., Alrfaei B.M., Halwani M.A. (2022). Viridiflorol induces anti-neoplastic effects on breast, lung, and brain cancer cells through apoptosis. Saudi J. Biol. Sci..

[83] Gautam R., Jachak S.M. (2009). Recent developments in anti-inflammatory natural products. Med. Res. Rev..

[84] Yuan G., Wahlqvist M.L., He G., Yang M., Li D. (2006). Natural products and anti-inflammatory activity. Asia Pac. J. Clin. Nutr..

[85] Aly S.H., Thabet A.A., Bahgat D.M., Mahmoud O.A., Elhawary E.A., El-Nashar H.A., Eldahshan O.A. (2026). Plant-Derived Compounds: A Potential Treasure for Development of Analgesic and Antinociceptive Therapeutics. Phytother. Res..

[86] Cao B., Xu Q., Shi Y., Zhao R., Li H., Zheng J., Liu F., Wan Y., Wei B. (2024). Pathology of pain and its implications for therapeutic interventions. Signal Transduct. Target. Ther..

[87] Silva-Correa C.R., Campos-Reyna J.L., Villarreal-La Torre V.E., Calderón-Peña A.A., Blas M.V.G., Aspajo-Villalaz C.L., Cruzado-Razco J.L., Sagástegui-Guarniz W.A., Guerrero-Espino L.M., Hilario-Vargas J. (2021). Potential activity of medicinal plants as pain modulators: A review. Pharmacogn. J..

[88] Turnaturi R., Piana S., Spoto S., Costanzo G., Reina L., Pasquinucci L., Parenti C. (2023). From plant to chemistry: Sources of active opioid antinociceptive principles for medicinal chemistry and drug design. Molecules.

[89] Mir M.A., Ashraf M.W., Singh P. (2020). The anti-diabetic and spectral analysis of various extracts of *Lilium polyphyllum*. Res. Sq..

